# Toward Low-Emission Agriculture: Synergistic Contribution of Inorganic Nitrogen and Organic Fertilizers to GHG Emissions and Strategies for Mitigation

**DOI:** 10.3390/plants14101551

**Published:** 2025-05-21

**Authors:** Shahzad Haider, Jiajie Song, Jinze Bai, Xing Wang, Guangxin Ren, Yuxin Bai, Yuming Huang, Tahir Shah, Yongzhong Feng

**Affiliations:** 1 College of Agronomy, Northwest A & F University, Yangling 712100, China; shahzadh@nwafu.edu.cn (S.H.); 18434763370@163.com (J.S.); jinze@nwafu.edu.cn (J.B.); wangxing0103@nwafu.edu.cn (X.W.); rengx@nwsuaf.edu.cn (G.R.); baiyx_@nwafu.edu.cn (Y.B.); huangyum@nwafu.edu.cn (Y.H.); 2 Shaanxi Engineering Research Center of Circular Agricultural, Yangling 712100, China; 3 College of Natural Resources and Environment, Northwest A & F University, Yangling 712100, China; tahir387@nwafu.edu.cn

**Keywords:** inorganic fertilizer, organic fertilizer, GHG emissions, CO_2_, CH_4_, N_2_O, emissions

## Abstract

Nitrogen (N) and organic-source fertilizers in agriculture are important to sustain crop production for feeding the growing global population. However, their use can result in significant greenhouse gas (GHG) emissions, particularly carbon dioxide (CO_2_), methane (CH_4_), and nitrous oxide (N_2_O), which are important climate drivers. This review discusses the interactive effects, uncovering both additive and suppressive outcomes of emissions under various soil and climatic conditions. In addition to examining the effects of nitrogen and the nitrogen use efficiency (NUE), it is crucial to comprehend the mechanisms and contributions of organic fertilizers to GHG emissions. This understanding is vital for developing mitigation strategies that effectively reduce emissions while maintaining agricultural productivity. In this review, the current knowledge is utilized for the management of nitrogen practices, such as the optimization of fertilization rates, timing, and methods of application, in terms of the nitrogen use efficiency and the related GHG emissions. Moreover, we discuss the role of organic fertilizers, including straw, manure, and biochar, as a mitigation strategy in relation to GHG emissions through soil carbon sequestration and enhanced nutrient cycling. Important strategies such as crop rotation, tillage, irrigation, organic fertilizers, and legume crops are considered as suitable approaches for minimizing emissions. Even with the progress made in mitigating fertilizer-related emissions, research gaps remain, specifically concerning the long-term effect of organic fertilizers and the interactions between microbial communities in the soil and fertilization practices. Furthermore, the differences in application practices and environmental conditions present considerable obstacles to accurate emission quantification. This review underlines the importance of conducting more thorough research on the combined application of N and organic fertilizers in multiple cropping systems to evolve region-specific mitigation strategies.

## 1. Introduction

Agriculture, forestry, and other land use (AFOLU) activities contribute approximately 13% of carbon dioxide (CO_2_), 44% of methane (CH_4_), and 81% of nitrous oxide (N_2_O) emissions from anthropogenic sources globally. These activities account for 23% (12.0 ± 2.9 GtCO_2_ eq yr^−1^) of the total net anthropogenic greenhouse gas (GHG) emissions [1]. CH_4_ and N_2_O have global warming potentials (GWPs) that are 27 and 273 times more prominent than CO_2_ over a GWP_100_, respectively, stepping up their environmental impact [2]. Furthermore, land use change (LUC) significantly impacts the global carbon cycle, contributing to elevated greenhouse gas (GHG) emissions and declining air quality [3,4,5]. Over the last 150 years, LUC has been responsible for approximately 35% of the total anthropogenic emissions [6,7]. Nevertheless, comprehensive data on the emissions from agriculture, forestry, and other land use sectors—such as residential, commercial, and recreational areas—remain scarce [8]. In recent years, agriculture has faced growing societal demands to increase protein production, further intensifying environmental pressures, with lower nitrogen inputs, ensure food security, and decrease GHG emissions simultaneously, while modifying itself toward challenges caused by a changing climate. In the last few years, substantial progress have been made in the area of food and agricultural production. As the population of the globe is likely to reach 10 billion by 2050, the effort to conserve finite assets like fresh water, cultivation land, and nutrients will increase. This highlights the instant need to adopt improvements in land use efficiency [9]. In agriculture, the prime nitrogen availability provides the biosynthesis of multiple non-protein compounds that are foundational to the physiological and metabolic processes of crops, as a result positively enhancing their yield and quality [10]. Nitrogen (N) makes up 0.1% to 0.6% of the soil weight in the top 15 cm, making it approximately 2000–12,000 kg-N ha^−1^, depending on the soil system characteristics [11]. Recent research emphasizes the trade-off between crop productivity and environmental sustainability associated with nitrogen fertilization. For example, nitrogen application resulted in only a 6% increase in the wheat yield, while the N_2_O emissions rose significantly by 73–245% compared to the unfertilized controls, indicating an optimal application rate of 180 kg N ha^−1^ [12]. Similarly, another study observed a 4–15% increase in the wheat yield with nitrogen fertilization at 270 kg N ha^−1^, which was accompanied by a 36–115% increase in the N_2_O emissions. These findings highlight the necessity of optimizing nitrogen inputs to balance yield objectives with greenhouse gas mitigation [13]. While nitrogen application at a rate of 112–119 kg N ha^−1^ increased the rice yield by 50%, it had no apparent impact on the global warming potential (GWP) or greenhouse gas intensity (GHGI) [14]. For sustainable agriculture, optimizing the N use efficiency (NUE) is important, because it provides a pathway to enhance fertilizer uptake and sustain crop yields under nitrogen-limited conditions. The latest advancements in molecular biology, agronomy, and plant physiology have positively enhanced our perception of N dynamics, paving the way for innovative strategies to enhance the NUE and build adoptable agricultural systems [15]. These existing systems, shaped by various anthropogenic land use practices [16], play a dual role in the environment by significantly contributing to global GHG emissions. They are responsible for approximately 14% of the total non-carbon GHGs, constituting a substantial 58% of these emissions [17]. These GHGs are playing a critical role in the contribution of global warming, which in turn leads to environmental losses such as soil degradation and climate change.

Research on the effects of combining organic and mineral fertilizers on soil fertility has shown that this approach boosts crop yields and soil organic carbon levels [18]. In light of the growing need to reduce agricultural GHG emissions from nitrogen fertilizers (NFs), a recent study has examined the impacts of NF on the crop yield and greenhouse gas emissions [18]. NF significantly increased the N_2_O and CO_2_ emissions by 43.8% and 10.6%, respectively, while only enhancing the tomato yield by 5.1% under 200 kg N ha^−1^ in spring greenhouse cultivation. Considering the minimal yield improvement and heightened emissions, it is recommended to adopt autumn cultivation together with 200 kg N ha^−1^ and aerated irrigation to reduce the greenhouse gas emissions and sustain the crop yield in the context of a warming climate [19]. However, both organic and inorganic fertilizers raised the N_2_O emissions, while the organic fertilizers emissions were comparatively less from inorganic sources [14]. A related study in sub-Saharan Africa demonstrated that the use of organic fertilizer sources resulted in significantly reduced emissions of N_2_O and CO_2_ compared to the application of solely mineral fertilizers [20]. These studies underline the best way to use nitrogen fertilization to boost the crop output and reduce the emissions of GHG, based on the crop kind, management of the fertilizer, soil characteristics, and climatic factors. The differences in these findings makes it difficult to offer universal recommendations for nitrogen fertilization management worldwide.

Straw return also has a complex impact on nitrous oxide (N_2_O) emissions and contributes to CH_4_ emissions, as evidenced by studies demonstrating varying outcomes [21]. Such emissions are initially affected by the processes of nitrification and denitrification, both responsive to environmental and management-related factors. For instance, the microbial breakdown of straw can produce anaerobic conditions by lowering the soil’s oxygen content, which would strengthen the denitrification activity and possibly increase the emission of N_2_O [22]. Considering the release of nitrogen during straw decomposition provides additional support for these processes, further contributing to the emission of N_2_O [23]. Though, the limits of these emissions vary, with variable factors including the soil type and nitrogen fertilizer application, plus the straw handling methods, positively affecting the outcomes [24,25]. In China, inappropriate straw management further contributes to these emissions, with activities such as open burning, heating and cooking linked to 446 million tons of GHG emissions annually [26]. Therefore, appropriate utilization of straw becomes a key tactic for lowering the greenhouse gas emissions from agricultural activities [27]. Studies show that carbon (C) sequestration through straw reincorporation provides a notable approach to lowering the emission of greenhouse gases [28,29]. Regardless of this, while analyzing the impact of straw return, it is also necessary to take into account agricultural management methods, which are essential to regulating CH_4_ emissions [30]. However, the implementation of large-scale straw return strategies necessitates a comprehensive assessment of their impact on soil greenhouse gas emissions, a topic that remains contentious [31,32]. Soil organic carbon (SOC) and the emission of CH_4_ are linked and even more apparent in studies reporting that returning straw frequently increases CH_4_ production because of the greater quantity of carbon in the soil and increases the activity of microbes [29,30,33,34]. Under favorable conditions, the procedure of incorporating straw into the soil goes through mineral formation and the process of humification, along with humus molecules formation [35]. This procedure boosts the soil organic carbon (SOC), a vital component of soil fertility that enhances crop production, soil aeration, and overall agricultural output [36].

Biochar, a byproduct of the high-temperature pyrolysis of straw, is globally recognized for its potential to enhance soil quality and sequester SOC, thereby playing a crucial role in reducing GHG emissions [37]. Its attributes include a polymeric laminar structure and a stable aromatic carbon skeleton, which are unsusceptible to degradation by soil microorganisms, thus rendering it an advantageous approach to climate change mitigation [35,38]. In the agricultural sector, the production of biochar through the pyrolysis of straw at very high temperatures has long been acknowledged as a dependable and effective method of preserving SOC [39]. Its characteristics mainly include a high porosity, a low bulk density, a large cation exchange capacity, and a strong aromatic carbon structure, and it also changes the microbial environment of the soil and affects the availability of the foundation for soil microbes. These characteristics carefully affect the formation and breakdown of various SOC components [21,40]. However, aging processes and other biotic and abiotic variables impact the long-lasting effects of biochar on SOC production, which makes necessary a more thorough knowledge of its function in preserving carbon [41]. In agricultural production systems, the carbon footprints (CFs) and nitrogen footprints (NFs) are commonly employed to address environmental effects [42]. Biochar amendments to soil have been found to lower the reactive nitrogen losses and GHG emissions, indicating substantial potential for improving environmental sustainability [43]. Such practices can lead to achieving the goals of mitigating environmental losses with balanced nutrient availability.

GHG emissions from manure occur from soil, emitting CO_2_ and CH_4_ processed by the degradation of organic matter, whereas N_2_O is first generated via the processes of nitrification and denitrification following manure application. N_2_O emissions may also occur laterally when nitrogen is removed through the volatilization of ammonia or nitrate leaching, which are subsequently converted into N_2_O [44]. Moreover, bacteria further decompose the complex organic compounds present in manure, leading to the production of CO_2_ in an aerobic environment [45]. However, the quantity of manure produced and the proportion that undergoes anaerobic decomposition significantly influence the CH_4_ emissions, which constitute a primary source of greenhouse gas emissions from manure [46]. Compared to non-manured soils, adding manure increases N_2_O emissions, necessitating viable manure management practices in the livestock industry. In contrast, synthetic fertilizers affect the root respiration and decomposition of microbes, particularly through the processes of nitrification and denitrification, thereby influencing the greenhouse gas emissions from the soil [47,48,49]. Therefore, the reduction of GHG emissions is crucial to ensuring the long-term sustainability of agricultural production systems.

Although numerous organic and inorganic strategies have been proposed to enhance crop yields and mitigate emissions, there remains a significant gap in the comprehensive understanding of the specific effects of these methods, particularly concerning the emission of nitrogenous gases associated with inorganic sources. Furthermore, practical strategies for the implementation of low-emission agricultural practices have not yet been thoroughly investigated. Low-emission agriculture is the major concern of the modern era, so this study reviews various agricultural approaches, as detailed in Table 1, to provide background information on organic methods for enhancing crop production to meet the demands of a growing population and their impact on greenhouse gas (GHG) emissions. By addressing GHG emissions from organic sources, this review offers clear insights into the mechanisms involved in nitrogenous gas emissions resulting from the application of inorganic sources and proposes practical strategies to achieve low-emission agriculture.

## 2. Methodology

For this review, research articles were sourced from the Web of Science database, with additional information obtained from Google Scholar and relevant applied reports familiar to the authors. For this purpose, a comprehensive search strategy was designed, combining various keywords related to both fertilizer application and greenhouse gas emissions, including methane, CH_4_, nitrous oxide, greenhouse gas emissions, N_2_O, CO_2_, carbon emission, fertilizer, emission, organic and inorganic nitrogen, straw, manure, compost, biochar, and application in the field. The search covered publications from 1916 to 2025 and was limited to peer-reviewed articles written in English. The titles, abstracts, and keywords were screened in the first round, and the full texts were reviewed for relevance in the second round. Studies that did not provide clear measurements of greenhouse gas emissions or lacked relevance were excluded. Overall, almost 710 articles were reviewed, with more than 245 ultimately being included in this review. The selected studies were further categorized based on the type of fertilizer (organic vs. inorganic) and reported emission metrics.

## 3. Inorganic Nitrogen Fertilizer with Associated N Losses

**Figure 1:**
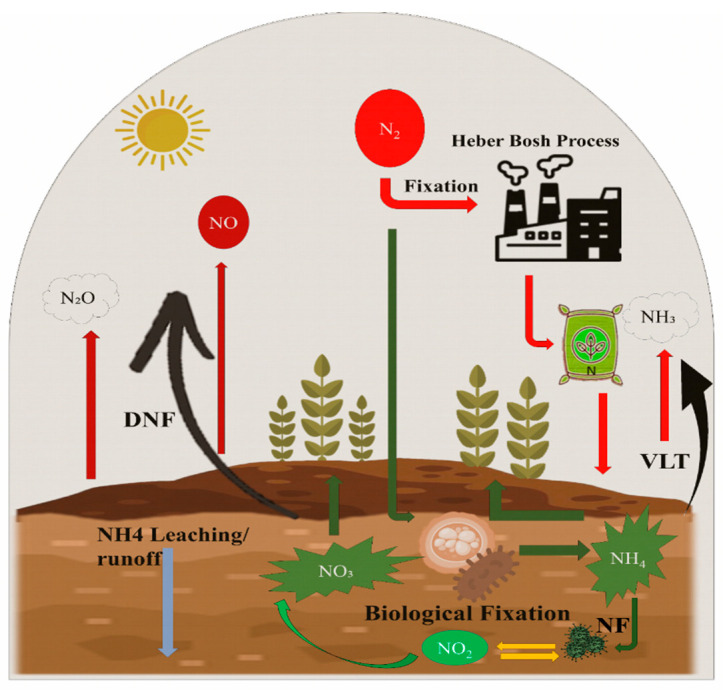
Soil nitrogen cycle associated with nitrogen fertilization along with the involved mechanisms.

Nitrogen (N) fertilizer production is mostly dependent on the Haber–Bosch process [56], which consists of a process that uses a lot of energy to make ammonia from hydrogen, which is usually made from nitrogen taken from the air and methane in natural gas. This ammonia provides the foundation for fertilizers like urea (CO (NH_2_)_2_) and ammonium nitrate (NH_4_NO_3_), which altogether account for roughly 75% of the global consumption of straight N fertilizers (International Fertilizers Industry Association) [57]. In the soil nitrogen cycle, N fertilization performs an important role, as described in Figure 1. In addition to synthetic nitrogen (N) fertilization, the biological fixation of inert atmospheric N into reactive forms and atmospheric deposition play crucial roles in the global nitrogen cycle.

The N cycle within agricultural soils emphasizes the interconnected pathways of nitrogen transformation and their environmental consequences [58]. Additionally, biological nitrogen fixation by soil microbes transforms N_2_ into ammonium (NH_4_^+^), thereby contributing to plant-available nitrogen [59]. However, nitrogen in the soil undergoes various transformations and losses. Nitrification and denitrification (DNF) processes result in the formation and emission of nitrous oxide (N_2_O) [60], a potent greenhouse gas [61]. Moreover, Figure 1 also illustrates volatilization (VLT), where NH_3_ is released into the atmosphere, and the leaching or runoff of NH_4_^+^, which can lead to water body contamination [62]. Furthermore, effective nitrogen management is crucial to balancing the crop yield with ecological sustainability [63].

Moreover, nitrogen fertilization (NF) has become a widespread agricultural practice used worldwide to increase crop yields; however, excessive application of nitrogen fertilization has led to significant GHG emissions, which is contributing in climate change and global warming [64]. According to a meta-analysis that examined factors like the crop productivity, greenhouse gas intensity (GHGI), and global warming potential (GWP) across 16 countries, the ideal rates of nitrogen fertilization are 130 kg ha^−1^ for rice, 180 kg ha^−1^ for wheat, 150 kg ha^−1^ for maize, and 200 kg ha^−1^ for vegetables or industrial crops [65]. Furthermore, the accumulation of nitrogen and its impact on greenhouse gas (GHG) fluxes in an alpine swamp meadow located on the Qinghai–Tibet Plateau were examined. It was found that nitrogen fertilization alone (4 g N m^−2^ yr^−1^) did not significantly influence the GHG fluxes. In contrast, warming alone (6.2 °C) resulted in a 30.9% increase in ecosystem respiration (Re), transformed the meadow from a sink to a source of N_2_O, and did not affect the CH_4_ flux. However, the combined effects of warming and nitrogen fertilization led to a 69.6% increase in CH_4_ uptake and a 26.2% increase in N_2_O emissions. These effects were modulated by seasonal variations, with the soil temperature affecting the Re, the soil moisture regulating the CH_4_ flux, and the rainfall influencing the N_2_O emissions. This study underscores the interactive effects of nitrogen and warming on GHG dynamics in alpine wetlands [66]. Additionally, a study conducted in China indicates that nitrogen fertilization significantly contributes to air pollution, with each gram of nitrogen fertilizer applied correlating with a 0.55 μg/m^3^ increase in the PM_2_._5_ concentrations. These effects vary according to the crop type: nitrogen application in rice and maize production notably elevates the air pollution levels, particularly during the early growth stages, whereas the impact on wheat remains less comprehensively understood. Furthermore, the application of nitrogen fertilizer tends to increase the PM_2_._5_ and SO_2_ concentrations, while concurrently reducing the ozone levels [67]. Moreover, based on a study in a Mediterranean maize system, conventional urea fertilization (130 kg N ha^−1^ yr^−1^) resulted in cumulative emissions of 2.2 Mg CO_2_-C ha^−1^ and 0.24 kg N_2_O-N ha^−1^, with an annual global warming potential of 194 kg CO_2_-eq ha^−1^ [68], as illustrated in Table 2. Furthermore, fertilizers such as urea, ammonium hydroxide, and ammonium bicarbonate lost more ammonia (NH_3_) through volatilization compared to fertilizers containing diammonium phosphate or ammonium sulfate [11]. However, the selection of the fertilizer type also affects the NH_3_ emissions, with urea and UAN fertilizers producing seven and four times higher NH_3_ emissions individually as compared to ammonium nitrate. Similarly, the NH_3_ volatilization losses are more considerable with urea, ammonium hydroxide and ammonium bicarbonate than with diammonium phosphate or ammonium sulfate [11]. Furthermore, the method, amount, kind, and timing of fertilizer application are also linked with the long-term fertility and health of soils. Studies have documented the adverse effects of prolonged or excessive use of synthetic fertilizers; for example, prolonged use of chemical nitrogen fertilizers has been associated with a reduction in the soil pH, irrespective of the application method. In contrast, the application of urea and animal urine has been observed to increase the soil pH [69].

### 3.1. Nitrogen Use Efficiency

For comparing different farming systems, farm nitrogen (N) measures are valuable mechanisms for comparing farm performance. The release of nitrogenous gases, including NO, N_2_O, and NH_3_, due to the losses caused by fertilizer nitrogen can remarkably lower the efficiency of nitrogen fertilization. The nitrogen use efficiency (NUE) and excess of nitrogen are the most commonly used indicators for addressing the impact of fertilization methods on the environment. These indicators can be calculated using Equations (1) and (2), respectively [74].

Nitrogen use efficiency (%) = N uptake/N fer × 100%(1)

Nitrogen surplus (kg Nper ha) = Ʃ(N inputs) − Ʃ (N outputs)(2)

Nitrogen management is a keystone of viable agricultural practices, equating the dual goals of production and environmental conservation. The nitrogen use efficiency (NUE) is crucial in this regard, representing the capacity of crops to effectively handle available nitrogen (N) resources while minimizing losses. The NUE can be obtained from a mass balance perspective, where the main components include nitrogen fertilizer application (Nfer, kg-N ha^−1^) and nitrogen uptake by crops (Nuptake, kg-N ha^−1^). Moreover, the nitrogen inputs (Ninputs, kg-N ha^−1^) encompass atmospheric deposition, surrounding fertilization, and irrigation water, while the nitrogen outputs (Noutputs, kg-N ha^−1^) refer to nitrogen removed through harvested crops and plant uptake. These factors indicate the significance of measuring the NUE alongside the nitrogen excess through inclusive crop analyses that include the nitrate content of crops. Recent methods such as handheld sensors have been developed to evaluate the NUE and its components more accurately [75]. Advanced studies have investigated strategies to improve the NUE through selection of cultivars, improved nitrogen application, and precise farming practices. For example, maize cultivar mixtures with interdependent root and leaf traits significantly enhanced the nitrogen application and NUE and provided higher grain yields [76]. The NUE by itself is a complex characteristic, having two key components: N uptake efficiency (NUpE) and N utilization efficiency (NUtE) [77]. Such factors are affected by the nitrogen supply change and are essential for defining the overall NUE. Efficient genotypes, for example, apply diverse morphological and physiological adaptations to enhance the NUE, allowing them to access and handle N resources more efficiently [78]. The fertilization practices prevalent in intensively cultivated wheat–maize systems, particularly in Northern China, are characterized by high nitrogen inputs to meet the high yield targets and specific soil conditions [79]. This is largely due to the excessive and prolonged use of nitrogen fertilizers, coupled with the low efficiency of farmer cultivation practices. Typically, 500 to 600 kg N ha^−1^ yr^−1^ of fertilizer is applied to achieve the maximum yield. Agronomically, this application rate is considered excessive when compared to the general crop nitrogen requirements, which range from 200 to 300 kg N ha^−1^ yr^−1^ [80], to achieve NUE, and to sustain over 90% of the maximum yield potential, applying an N rate of 420 kg N ha^−1^ yr^−1^ is ideal, and resultantly, it will reduce greenhouse gas (GHG) emissions to 1.15 t CO_2_-eq ha^−1^ yr^−1^. Accommodating nitrogen inputs seasonally lowers the emissions further to 1.07 t CO_2_-eq ha^−1^ yr^−1^ without effecting the yield, enhancing the effectiveness of calculated nitrogen management in intensive agriculture [81]. Achieving a high NUE is crucial in high-input cropping systems, where excessive nitrogen applications raise environmental concerns. Over 90% of the total N_2_O emissions are caused by excessive nitrogen use, particularly at rates exceeding 550 kg N ha^−1^ yr^−1^ [82,83]. In response to this, sustainable agricultural practices have been developed, including techniques such as deep fertilization [84], use of slow-releasing fertilizers [85], fertilization via biochar [86], and modified-clay composites [87]. This revolution not only improves the NUE but also decreases the nitrogen surplus and nitrogenous gas emissions while preserving high crop yields [88].

### 3.2. Emissions Associated with Nitrogen

The implementation of N fertilizer is a crucial agricultural practice for improving rice yields, although significant environmental issues are linked with it, especially ammonia (NH_3_) volatilization and GHG emissions. It was noted that throughout the growing season for rice, the collective NH_3_ emissions were substantially (*p* < 0.05) increased by 22.60–25.55% [89]. However, the environmental concerns related to improper or excessive N fertilizer application expand beyond GHG emissions. Such methods guide decreased nitrogen utilization rates, soil nutrient variance, acidification, water pollution, and salinization, along with high emissions of NH_3_, NO, and other related gases [90,91,92,93]. NH_3_ evaporation in paddy fields is mostly determined by the kind and amount of N fertilizer used, which affect the NH_4_^+^ aggregations and related vaporization rates [94,95]. NH_3_ that is volatilized from the fertilized fields is the major source of atmospheric NH_3_ and ammonium (NH_4_^+^), which can counter sulfur dioxide (SO_2_) and NO_x_ to make secondary molecules such as ammonium sulfate and ammonium nitrate [96]. Therefore, NH_3_ is taken to be one of the supremely responsible ingredients in terms of the formation of atmospheric aerosol [92]. The process linked with NH_3_ volatilization is rapid, normally occurring within one week of N fertilizer application. This procedure is directly affected by the soil’s physical and chemical properties, such as the cation exchange magnitude, pH, and texture, which influence the intake of NH_4_^+^ by soil colloids and its transformation into NH_3_ [97]. Moreover, components such as the temperature, timing and type of N fertilizer applied are indirectly affecting the volatilization rates [98]. An elevation in temperature due to GHG emissions also has an impact on NH_3_ volatilization, creating a feedback loop that worsens environmental impacts [99]. Simultaneously, the greenhouse gas (GHG) emissions, including those comprising N_2_O, from paddy fields are significantly influenced by the nitrogen cycle, thereby substantially contributing to global warming [100]. Additionally, N fertilizer application improves growth and enhances the input of exogenous carbon (e.g., root exudates, litter, and stubble) [101], while excessive nitrogen application leads to environmental degradation, contributing to groundwater contamination and increased N_2_O emissions, which exacerbate climate change. To achieve carbon neutrality by 2050 and limit global warming to 1.5 °C above preindustrial levels, it is imperative to reduce anthropogenic greenhouse gas (GHG) emissions—including N_2_O—by 43% from the 2019 levels by 2030 [102].

**Figure 2:**
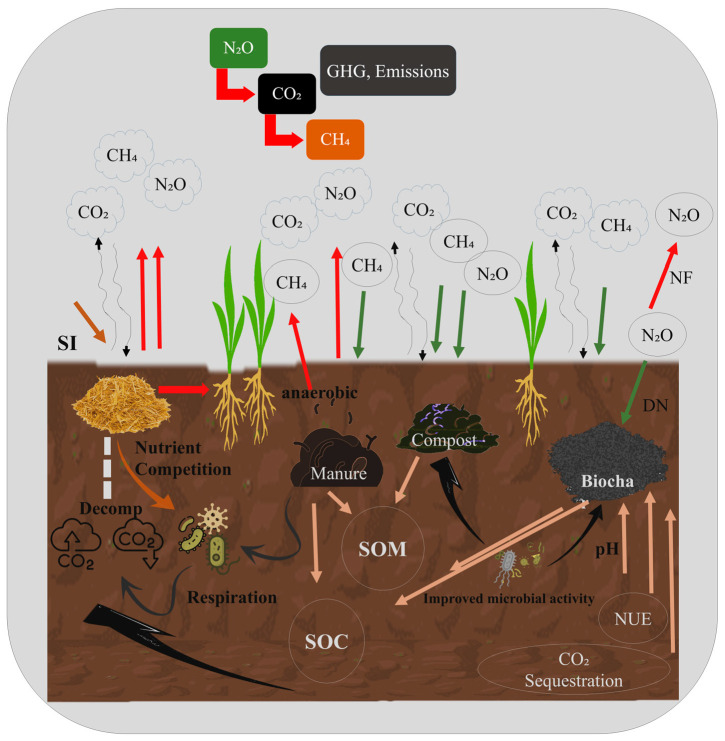
Illustration of various GHG emissions from organic fertilizers, which are straw, biochar, manure and compost.

Figure 2 emphasizes the key GHGs—carbon dioxide (CO_2_), methane (CH_4_), and nitrous oxide (N_2_O)—which originate from diverse sources, such as soil respiration, decomposition, and anaerobic conditions. Organic inputs, including straw incorporation (SI), manure, compost, and biochar, play a crucial role in altering soil processes [103,104]. For example, straw and manure enhance microbial activity and nutrient competition, potentially increasing CO_2_ and CH_4_ emissions under specific conditions [105,106]. Conversely, compost and biochar contribute to the accumulation of soil organic matter (SOM) and the storage of soil organic carbon (SOC), thereby facilitating CO_2_ sequestration [107]. Biochar also enhances the soil pH and microbial activity, while reducing CH_4_ emissions and modifying microbial pathways [108,109]. Anaerobic zones promote CH_4_ production [110], whereas denitrification (DN) leads to increased N_2_O release [111]. Figure 2 depicts the intricate interactions among soil amendments, microbial dynamics, and nutrient flows in the regulation of greenhouse gas emissions and the enhancement of soil health.

## 4. Straw Incorporation’s Impact on Greenhouse Gas Emissions

Straw incorporation significantly contributes to the enhancement of soil organic carbon (SOC), as illustrated in Figure 2, serving as a sustainable agricultural practice [112,113]. This approach also improves the nutrient availability [114] and soil structure [115]. Moreover, incorporating straw along with chemical fertilizers during the fallow period resulted in a 144% increase in methane emissions and an 80% higher global warming potential compared to conventional practices, although it effectively reduced the nitrous oxide emissions during the subsequent maize cultivation phase, as described in Table 2 [75]. In recent decades, approximately 47% of straw has been reincorporated into soils globally [116]. This practice is primarily driven by two major objectives: promoting sustainable agriculture to ensure food security and mitigating climate change. Consequently, straw incorporation is widely recognized as an effective field management strategy. However, the increase in the carbon-to-nitrogen (C:N) ratio presents certain challenges [117]. A high C and N ratio can accelerate severe outcomes, like competition for nutrients between crops and soil microorganisms (described in Figure 2) [118], and steady decomposition rates [119] can lead to an increase in N_2_O and CO_2_ emissions by 28% and 32%, respectively, over the following six years [31]. Additionally, straw incorporation is widely recognized as an effective method for enhancing soil fertility; however, it substantially increases CH_4_ and N_2_O emissions [120]. Furthermore, the retention of crop residues across China’s agricultural lands contributes to increased crop yield, soil organic carbon, nutrient availability, and moisture. However, it also leads to a significant rise in emissions of CO_2_ (31.7%), CH_4_ (130.9%), and N_2_O (12.2%), as described in Table 3 [121]. Moreover, a study based on a meta-analysis also observed more GHG emissions from straw incorporation as compared to no straw being applied [112].

Besides increasing GHG emissions, the addition of straw also influences soil carbon sequestration by directly boosting the carbon input, thereby improving the root residue accumulation [122,123]. Short-term variations in carbon are unaffected, as demonstrated by continuous measurements of the soil organic carbon (SOC) content [124,125]. For assessing seasonal or annual variations in soil carbon, the net ecosystem carbon budget (NECB) has gained widespread acceptance as an advanced method [126,127,128]. The NECB restores equilibrium between the ecosystem’s carbon inputs and outputs, providing awareness of the system’s extent for carbon utilization [129]. In addition, the NECB, along with CH_4_ and N_2_O emissions, is included in the overall greenhouse gas budget (NGB) by converting them into CO_2_ equivalents using the global warming potential (GWP) coefficients, permitting an absolute evaluation of the warming effect of agricultural practices. Most studies have focused on GHG emissions using the GWP [120,128]. In the context of sustainable agriculture, the assessment of the net ecosystem economic benefits (NEEBs), which involves balancing crop yields, agricultural costs, and global warming potential (GWP), is crucial for aligning economic and ecological objectives [30]. Furthermore, the incorporation of straw into soil enhances carbon storage by converting CO_2_ into stable carbon pools, which may mitigate the rapid increase in greenhouse gas emissions through the utilization of straw [126]. Moreover, a long-term field experiment expressed the lowest net GHG emissions and increased net ecological economic advantages, highlighting its potential as a low-impact and high-output strategy through straw-returning. It was revealed that the total carbon and *nirK* (a gene found in soil bacteria involved in denitrification) abundance is an important forecaster of the GWP during the booting and maturity stages of rice growth by using the random forest model. The study emphasized the value of balancing straw utilization to optimize agricultural productivity and environmental sustainability [130]. Alongside straw application affecting microbial growth, enhancing nitrification and denitrification processes, which increase N_2_O emissions [96,131], few studies have inspected the relationship between carbon sequestration and GHG emissions, mainly in evaluating the impact of the assimilated straw C:N ratios on the NGB. Including the GHG emissions respective to the grain yield through metrics such as the net greenhouse gas intensity (NGHGI) delivers a clearer understanding of the environmental values of production [132].

**Table 3:** GHG emissions by various fertilization methods.

Management Practice	N_2_O Emission	CH_4_ Emission	CO_2_ Emission	References
Nitrogen (DN600) + (conv. N) + N-enriched	↓21% to ↑90%	↓21% to ↑90%	↑10.6% to ↑40%	[133,134]
Straw (crop residue)	↑12.2%	↑130.9%	↑31.7%	[121]
Biochar (obtained from sugarcane) + CS	↓27.7% to ↓71%	↑15%	↑16% to ↑70%	[109,135,136]
Manure (chicken and horse manures) + Am + cow manure (lactating and dry cow) + VRM + CRM	↑25.8%	↓25% to ↓85%	↑45%	[137,138,139,140]

## 5. Manure as a Substitute Approach

Recently, considerable attention has been directed toward understanding how manure management impacts both direct and indirect sources of greenhouse gas (GHG) emissions. This interest is primarily due to the substantial amounts of nitrogen—often in inorganic forms—and organic carbon present in manure, which are critical factors influencing the microbial processes that result in emissions of nitrous oxide (N_2_O) and methane (CH_4_) [141,142,143]. While manure is a recognized contributor to GHG emissions, the management practices employed can significantly influence the magnitude of these emissions. Consequently, appropriate management strategies can mitigate gaseous losses [106]. Nitrous oxide emissions predominantly arise from nitrification and denitrification processes in soils following manure application [144,145,146,147]. Additional emissions may originate from livestock bedding, solid manure heaps [148,149], and the surface layer of slurry storage [150]. In fresh manure and slurry, inorganic nitrogen is primarily present as ammonium (NH_4_^+^), which can be oxidized to nitrate (NO_3_^−^) through nitrification, producing N_2_O in the process. This NO_3_^−^ subsequently serves as a substrate for denitrification, potentially releasing further N_2_O when the process is incomplete [106]. Methane emissions, primarily originating from enteric fermentation and flooded rice fields, can also be produced from manure during the anaerobic decomposition of organic matter in feces and bedding [142,151,152]. Under anaerobic conditions, acid-producing bacteria decompose organic matter into volatile fatty acids, which methanogens subsequently utilize to generate CH_4_. Factors such as the temperature, the composition of organic material, and the manure management strategies significantly influence CH_4_ production [153,154].

In agricultural systems, manure is extensively employed as a nutrient-rich fertilizer with the potential to enhance soil fertility and promote carbon sequestration [155]. However, its impact on soil carbon storage and nutrient cycling is heavily dependent on the manure quality and composition, which remain subjects of ongoing research [156]. The application of animal manure to croplands is regarded as an environmentally sustainable practice, offering advantages such as improved soil fertility [157,158,159] and increased soil organic carbon, as shown in Figure 2 [160,161]. These benefits may arise both directly, through the input of organic matter, and indirectly, by enhancing microbial activity and nutrient retention. Furthermore, manure application can elevate the soil pH [162,163], a critical factor influencing N_2_O emissions. Research suggests that a higher soil pH can lead to a significant reduction in N_2_O release [164,165], underscoring the importance of pH regulation in greenhouse gas mitigation strategies [166].

The use of organic fertilizers, such as animal manure, as a partial substitute for mineral fertilizers (MFs) is regarded as a beneficial strategy to reduce MF usage and enhance both soil organic matter and nutrient cycling in agricultural systems. However, it is imperative that the implementation of this approach does not result in any adverse environmental conditions [167,168]. It was evaluated in a recent study that N_2_O and CO_2_ emissions were also observed by replacing MF with various animal manures as compared to replacement with PM and solid cattle manure, where CsM decreased the CH_4_ emissions [167]. Additionally, a comparative study has demonstrated that manure significantly enhances nitrogen availability, crop productivity, and greenhouse gas emissions. Specifically, chicken manure was found to increase N_2_O emissions and CO_2_ emissions by 45% compared to horse manure, as described in Table 3. This finding highlights the critical need for optimized manure management strategies to balance agronomic benefits with the mitigation of greenhouse gas emissions [137]. Additionally, manure application enhanced the soil’s nutrients and organic matter [169,170]. This effect is especially important in Mediterranean regions, where soils are often characterized by low organic matter content and are susceptible to erosion [171,172].

The application of animal manure in Europe is a strongly proposed set of operations for modifying the nutrient cycles in agriculture by rehabilitating it as crop fertilizer [173]. Such adaptation lowers the reliance on mineral fertilizers and improves sustainable economy in agriculture and reduces their associated impacts. However, manure can take part in the production of GHG with the application of nitrogen (NH_4_^+^-N and NO_3_^−^-N) [106,174]. Due to ongoing concerns about global warming and climate change, researchers are focused on sustainable adaptations related to all human activities. The primary cause of environmental problems in the past few years is thought to be the increase in GHG emissions, a trend in which food production methods are heavily involved [175]. GHG emissions and reactive N emissions from croplands are considered to be because of long-term excessive N application. A comparative analysis revealed that substituting nitrogen fertilizer with cow manure at ratios of 20.00% (CD20) and 50.00% (CD50) yielded favorable outcomes, as evidenced by field research conducted over four consecutive cropping seasons. Specifically, the CD20 treatment resulted in a 6.65% reduction in N_2_O emissions and a 48.08% increase in the soil organic carbon (SOC) concentration, while maintaining yield levels. However, when compared to conventional nitrogen application (CK), the greenhouse gas (GHG) emissions and greenhouse gas intensity were elevated by 36.13–36.44%. The 50.00% substitution ratio (CD50) led to an 8.77% reduction in the crop yield but resulted in a substantial increase in the SOC concentration, which rose by 295.82%, attributable to the higher N fertilizer substitution. These findings highlight the influence of varying manure substitution rates on the emissions and soil carbon dynamics in sweet maize cultivation, as shown in Table 2 [71].

## 6. Biochar Amendment and Its Attributes Toward GHG Emissions

Biochar, a carbon-rich substance produced by biomass pyrolysis or the burning of crop residues in an oxygen-deficient environment, is currently being considered as a promising strategy to lower nitrous oxide (N_2_O) emissions [37]. Considering the study by the Intergovernmental Panel on Climate Change, biochar has been embraced as a mitigating alternative known for its capacity to lower emissions and regulate land use [176]. Biochar’s characteristics, including its specific surface area, porosity, pH, acidic/basic functional groups, and redox characteristics, are mostly determined by the pyrolysis conditions and raw material, which might affect the formation of N_2_O [177]. Biochar, produced through pyrolysis, is a highly stable form of carbon. It can increase the soil organic matter, total nitrogen levels, and soil microbial diversity [178]. Biochar is more resistant to microbial decomposition than the original co-products [179], and it is suggested that it can persist in soil for hundreds to thousands of years [21,180]. Yet, recent research has highlighted its fragile and weak physical characteristics, inducing the breakdown into bioavailable colloidal fractions [181]. In comprehensive meta-analyses, it was demonstrated that biochar application led to average depletions in soil N_2_O emissions of 49% [77] and 38% [182] in laboratory and field experiments simultaneously, despite the fact that in some studies no effect or even an increase in N_2_O emissions was observed [183]. The relation between soil and biochar can change the nitrogen transformation by disturbing the soil pH, the activity and formation of soil microbes, and aeration [184], which may improve crop growth and enhance soil quality [185]. Moreover, biochar application may enhance nitrogen take up and utilization by plants, notably enhancing its physiological activity, determining the internal nitrogen use efficiency [186]. For example, crop-residue-derived biochar could sequester up to 3.7 gigatonnes of CO_2_ equivalent per year [187]. One prime strategy for assessing biochar’s CO_2_-limiting ability is carbon crediting, a positive tool to cope with and manage global greenhouse gas (GHG) emissions [188]. A long-term field experiment on a rice–wheat rotation system in eastern China revealed that the application of biochar from straw into the field (BCF) significantly mitigated the greenhouse gas emissions compared to straw from the crop into the field (SCF). During the rice growing season, BCF reduced the CH_4_ and N_2_O emissions by 58% and 25%, respectively, in comparison to SCF (Table 2). Although BCF led to a marginal increase in N_2_O emissions during the wheat season, it achieved a 51% reduction in the annual global warming potential (GWP). Both SCF and BCF sustained the rice yields comparable to those achieved with chemical fertilizer alone (CF), with BCF causing a slight, non-significant decrease in the wheat yield. These results suggest that converting straw to biochar prior to soil application is more effective in reducing greenhouse gas emissions than direct straw incorporation, while maintaining crop productivity in rice–wheat rotation systems [70]. Furthermore, the incorporation of 1.9% sugarcane-derived straw biochar during wheat cultivation resulted in a 16% increase in the shoot biomass and a 27% enhancement in the grain yield. The soil amended with biochar emitted less N_2_O compared to the control (without biochar), indicating improved nitrogen use efficiency. The enhanced nitrogen uptake and its effective conversion into the grain yield underscore biochar’s potential to facilitate sustainable crop production while mitigating N_2_O emissions from nitrogen fertilization in tropical conditions [148,189]. Because biochar contains a lot of refractory carbon, which is crucial for carbon sequestration, it has the potential to slow down climate change [127,190].

The use of biochar to capture CO_2_ is one possible mitigation technique to reduce atmospheric CO_2_ levels in the face of ongoing variations in the global temperature and atmospheric carbon balance [40,191]. As can be seen in Table 2, biochar-based soil additions may lower greenhouse gas emissions [191]. Moreover, a limitation in terms of CO_2_ production has also been noted [39,192]. Comparatively, other examinations reported no important variations in or even encouragement of CO_2_ production [193]. Correspondingly, the CH_4_ emissions have been reduced in some cases [194] but raised in others [195]. Biochar at the time of pyrolysis possibly sequesters nitrogen inside its aromatic structure [196,197] and after soil applications reduces N_2_O production [198]. Therefore, biochar can serve as a slow-release nitrogen fertilizer and may act as a medium to deal with nitrogen losses in agricultural soils [199]. It has been assumed that biochar is lowering N_2_O emissions in soils that have high denitrification activity but possibly influencing N_2_O emissions in soils by enhancing nitrification processes [77]. It is unclear what mechanism underlies these reductions and how much of them occurs in soils [200]. The difference in GHG output by biochar amendments is caused by a number of reasons, such as the diversity in biochar properties being a factor contributing to the discovered variations. As biochar production relies on the pyrolysis conditions, residual material, and post-production means, it has multiple characteristics [201,202]. These differences may impact the microbial communities, nutrient accessibility, and soil physical and chemical characteristics, all of which may have an impact on GHG emissions.

## 7. Strategies to Mitigate GHG Emissions

Global climate change is causing significant challenges to agricultural productivity [203]. At the same time, agricultural systems are also contributing to atmospheric GHG emissions, which are causing climate change [204]. The primary cause of global warming is the increase in GHG emissions, particularly N_2_O and CO_2_, which have risen by 20% and 40%, respectively, due to global development [205]. Studies have estimated that agricultural lands account for approximately 25% of global GHG emissions [206], making it a major contributor to these emissions [207]. Mitigating GHG emissions in agriculture necessitates the implementation of integrated and sustainable management practices [208]. Agronomic adaptation, which encompasses adjusting planting dates, selecting climate-resilient crop varieties, and employing conservation tillage, can reduce soil disturbance and enhance carbon sequestration, thereby lowering CH_4_ and N_2_O emissions [209,210]. Enhancements in irrigation and nitrogen fertilization, including the use of precision irrigation systems and optimized nitrogen application strategies, further improve the nitrogen use efficiency and limit nitrogen losses [211]. Additionally, crop rotation with legumes provides an effective biological alternative to synthetic fertilizers by fixing atmospheric nitrogen, improving soil fertility, and reducing nitrogen losses [64]. Moreover, the application of organic sources of fertilization supports soil health, promotes long-term carbon storage, and reduces reliance on chemical fertilizers [212]. When applied appropriately, these strategies synergistically contribute to reducing agricultural GHG emissions while maintaining crop productivity.

### 7.1. Agronomic Adaptation

**Figure 3:**
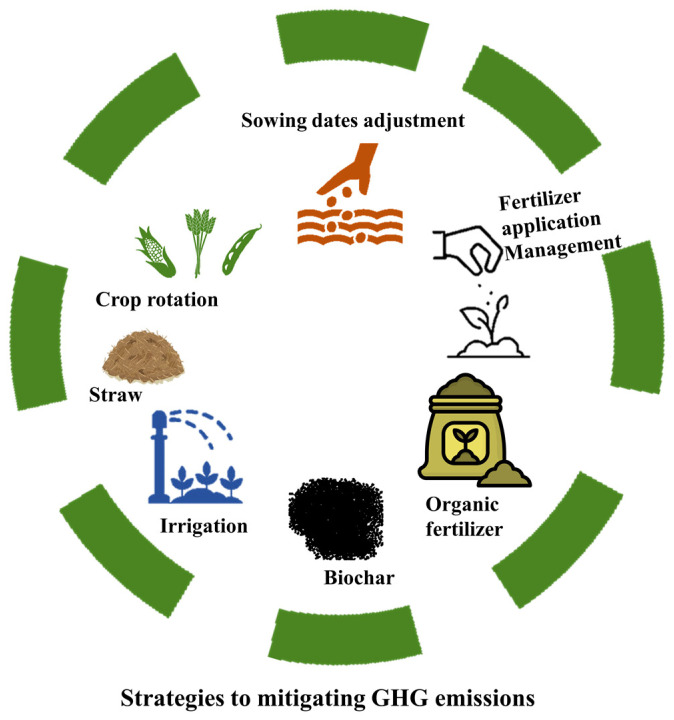
Possible strategies toward low-emission agriculture.

To introduce functional crop management modification strategies to deal with climate change, the relationship between crop growth, management practices, and environmental conditions is required to be considered [213]. In response to the challenges posed by climate change, numerous agronomic strategies have been proposed to sustain global and regional crop production. These strategies include the development of new crop varieties, the implementation of crop rotation systems, the adoption of precision agriculture techniques, the adjustment of sowing dates, the enhancement of irrigation methods, and the revision of fertilization practices [214,215]. Among these strategies, adjusting irrigation and fertilizer application rates, as well as the timing of sowing, represent cost-effective, farm-based policies with promising potential for implementation without necessitating significant technological advancements. For example, phenological development can be stimulated by adjusting the planting dates in response to temperature variations to enhance crop vigor [216], as illustrated in Figure 3. In terms of the water use efficiency, it is imperative to improve irrigation practices and monitor increased evapotranspiration during the growing season [217]. Moreover, to enhance crop growth and mitigate the dilution effects on crop tissues, increased fertilization rates may be necessary under elevated CO_2_ conditions to maintain the quality of the harvested crops [218,219]. Even so, the majority of the research studies are focusing on agronomic adaptation to enhance crop production, and the output of these measures on agricultural soils’ existing greenhouse gas (GHG) balance has largely gone unnoticed [215]. Agronomic adaptation strategies can play a vital role in climate change, which is a significant risk for agriculture to sustain crop production with low GHG emissions. These strategies can be early sowing, an adjusted amount of fertilization, crop rotation and modified irrigation. An experiment conducted in Denmark revealed that a diversified crop rotation system, comprising potato, winter wheat, spring barley, and faba bean, resulted in an average total yield of 29 t DW ha^−1^ and soil greenhouse gas emissions of 3.02 t CO_2_e ha^−1^. Increasing the fertilization rate with early sowing lowered the yields by 6.1% and 4.8% subsequently. Though, incorporating early sowing with more irrigation or both irrigation and fertilization resultantly increased the yield up to 2.3% and 4.0%. Such strategies may increase soil GHG emissions by 4.1% to 17.8%, with elevated emissions noted when early sowing, fertilization, and irrigation were combined [220]. The soil moisture is a critical factor influencing N_2_O emissions from the soil through its impact on soil respiration, nitrification, denitrification, and mineralization processes. N_2_O emissions are predominantly regulated by denitrification, which occurs following irrigation [221] or rainfall [222]. This highlights that although agronomic adaptations may support food production under climate change, they could also contribute to GHG emissions, emphasizing the need for sustainable approaches in agriculture [220]. Though, limited research is available on the impact of agronomic adaptation measures on greenhouse gas emissions from soil, mostly concerning the N cycle [223,224].

### 7.2. Improvements in Irrigation and Nitrogen Fertilization

Agricultural soils are recognized as significant contributors to greenhouse gas emissions, particularly nitrous oxide (N_2_O) and carbon dioxide (CO_2_) [225]. Numerous studies have examined the influence of fertilizers and water on the transformation of carbon and nitrogen in soils, which can subsequently affect crop yields and emissions of greenhouse gases such as CO_2_ and N_2_O [226,227]. Furthermore, in the conventional urea (CONV) treatment, considered to be the baseline in the study, the total annual GHG emissions were approximately 194 kg CO_2_ eq ha^−1^ yr^−1^, with higher early-stage N_2_O emissions during the maize period (0.24 kg N_2_O-N ha^−1^) and notable CO_2_ emissions (2.2 Mg CO_2_-C ha^−1^), highlighting the environmental footprint of typical urea-based fertilization in drip-irrigated maize systems [68], as addressed in Table 2. Enhancing the crop yield while mitigating the greenhouse gas (GHG) emissions from agricultural practices has been a persistent objective. However, the demand for increased food production often conflicts with environmental conservation efforts [228]. The primary challenges are attributed to the excessive use of fertilizers and the scarcity of water resources [229]. In the North China Plain, the crop yield is significantly improved through substantial inputs of irrigation water and chemical fertilizers [230]. Additionally, in the wheat–maize rotation system of northern China, the substitution of flood irrigation with drip fertigation (DN600) resulted in a 21% reduction in N_2_O and CH_4_ emissions (shown in Table 3) and improved soil carbon sequestration [133]. Moreover, to reduce GHG emissions from soil, it is crucial to apply fertilizers judiciously and manage water resources efficiently. GHG emissions resulting from the application of inorganic nitrogen fertilizers and various irrigation strategies may have beneficial effects [230] and could serve as a strategy to regulate the increase in atmospheric GHG concentrations [231]. It has been well established that the soil moisture amount directly affects the GHG emissions from agricultural lands [232]. Among various agronomic practices, the irrigation technique is among the most important practices that directly affect the nitrogen and carbon turnover process. Also, irrigation is an important practice in agricultural systems, especially in arid and semi-arid areas where, without irrigation, increasing crop production and achieving higher productivity are not possible [233]. The amount of irrigation water applied could have a substantial influence on the intensity of the emission event. Research studies have shown that the timing and amount of irrigation water applied to crops could play a role in reducing GHG emissions [234]. An increase in temperature may expedite the decomposition of soil organic carbon (SOC) and enhance N_2_O production by promoting denitrification and mineralization processes [235], while variations in precipitation patterns may improve the soil moisture, certainly directing it toward higher SOC from escalated net primary production and elevated N_2_O emissions from increased nitrogen turnover [236]. Changes in the climate may also change the crop biomass progress, influencing soil GHG emissions along with changes in the nutrient absorption as well as the amount and properties of residues, which affect N and C fluxes [237]. Enhancements to the process of fertilization and irrigation providing moisture and base availability for denitrification are expected to influence N_2_O emissions through soil condition changes [238]. The impact of deficit irrigation on N_2_O emissions from wheat fields was further assessed on the North China Plain. Compared to flood irrigation, deficit irrigation can lower N_2_O emissions and increase the water usage efficiency [239,240]. Nitrogen implementation is considered the main agricultural practice contributing 30–50% of crop yields, which is playing a vital role in boosting the yield [241]. Overuse of nitrogen in crop production is causing serious ecological and environmental problems [24,242]. According to studies, N fertilizer contributes 36–52% of the overall agricultural GHG emissions [243]. It is suggested that, to sustain the yield with decrease in the N_2_O emissions by about 31%, the nitrogen input should have to be reduced by 28% [244,245]. Additionally, to sustain the crop growth and water use, balanced nitrogen application can be a suitable option [112]. Significant results have been obtained in summer maize at various growth stages in order to meet crop nutritional needs and achieve low carbon emissions [112]. However, studies also used different growth phases to evaluate the effect of nitrogen fertilizer on greenhouse gas emissions [246]. Resultantly, the study explored the impact of irrigation and optimal fertilization during the growing seasons on maize production and greenhouse gas emissions [247]. By optimizing the irrigation and nitrogen management, both the yield and environmental sustainability can be address. It was discovered that the summer maize greenhouse gas emissions were influenced by the nitrogen fertilization and irrigation at various development stages. The CO_2_ emissions were noted to be elevated in the ear stage, with nitrogen fertilization and irrigation contributing notable impacts on the global warming potential (GWP). Lowering the nitrogen (N1) led to an 8.88–13.3% lower GWP as compared to conventional nitrogen fertilization. Reducing the irrigation with conventional nitrogen in normal and wet years, lowered the GHG emissions and improved the yield as compared to the prevailing irrigation, with the limitation in nitrogen dosage showing a 29.3% GWP reduction in dry years [248]. Meanwhile, the cropland GHG emissions were regulated by the soil moisture produced by irrigation or precipitation [249]. To lower the GHG emissions from farmland and to enhance the nitrogen availability during the overall growth period, suitable pre-sowing irrigation is suggested [250].

### 7.3. Rotation with Legume Crops

Crop rotation, a well-established agricultural practice, entails the systematic alternation of crops on a single plot, yielding benefits that extend beyond mere productivity. These benefits include enhanced soil health, improved nutrient cycling, effective pest management, and a reduction in chemical usage. Diversified cropping systems have the potential to significantly decrease greenhouse gas (GHG) emissions, thereby contributing to climate change mitigation. The rice–wheat–green-gram system exemplifies resource optimization and minimizes chemical inputs, resulting in reduced GHG emissions [251]. Furthermore, crop rotation can substantially lower GHG emissions and enhance soil health. By alternating between water-intensive rice, water-efficient wheat, and leguminous green gram, farmers can optimize resource utilization and decrease chemical inputs, thus reducing GHG emissions [252]. Rotation systems are widely implemented in agriculture to boost productivity and mitigate soil degradation [253]. Crop diversification holds considerable promise for enhancing yield productivity while simultaneously improving ecosystem services, such as pest and disease control, carbon sequestration, and soil fertility [254,255]. The inclusion of legumes in the rotation is a pivotal strategy for sustainable agriculture [256,257]. Legumes fulfill crop nitrogen requirements through biological nitrogen fixation, supplying nitrogen to subsequent crops and thereby reducing the reliance on synthetic nitrogen fertilizers [258,259]. The cultivation of grain legumes not only bolsters soil health [260] but also supports sustainable crop production [261,262]. Generally, legume-based rotations result in lower N_2_O emissions compared to cereal-based systems [263]. Rotation cycles like legume–cereal have been important in maintaining viable agriculture because of legumes’ nitrogen-fixing ability [264,265], and while these rotations are known for lowering fertilizer N use and improving sustainability [261], their acquisition remains limited even with the growing need for legumes with grain [266]. Studies from China recommended that rotations based on legumes, such as soybean–maize and soybean–wheat, can boost the nitrogen use efficiency (NUE) and decrease emissions related to GHG, as described in Table 4 [267,268]. Synthetic N fertilizer causes N_2_O emissions through application and CO_2_ emission via intensive energy production [269]. Studies have examined how ammonia (NH3) emissions are caused by synthetic N fertilizer application having significant impacts on the environment, such as acidification and eutrophication [270]. Many studies across various climate zones suggested lowering the N fertilizer needs by integrating grain legumes into crop rotations, which reduces the environmental effect of these production methods during the year of legume production and subsequent years by incorporating grain legumes into crop rotations [271,272]. Yet, the next crop must efficiently consume the nitrogen that is provided to the soil by the atmospheric biological fixation of the legumes [273].

A reduction in the dependency on synthetic N fertilizer can be achieved by biologically fixed N via legume–rhizobia symbioses alternately reducing the associated GHG emissions. N_2_O emissions can be caused by adding the above-ground residue parts of legume crops to the soil [258]. Mostly, it is based on the quality of the residual biomass and total N_2_ fixed by legumes, specifically the ratio of C and N. Residues of legume like green manures, having a low C and N ratio, generally elevate N_2_O emissions because they provide both an unstable C substrate for NO_3_ denitrification and a source of N for nitrification, which is demonstrated in Figure 2. Higher C:N ratios are often not a significant source of N_2_O emissions and are observed in legume residues, such as senesced residues following harvesting pulse, due to slowed N release or possibly soil NO_3_ immobilization [258]. There are few studies available that distinguish between rotating cereal with a pulse crop versus single fertilized cereal to measure its impact on GHG emissions. The dose of N fertilizer was kept constant to assess the remaining N from the next legume crop, even though the same quantity of N was administered, and after a pulse crop, lower or comparable N_2_O emissions were observed in both cropping systems [279,280,281]. Thus, the fertilized cereal crop was be prone to GHG emissions compared to the crop rotations’ legume phase, which can help reduce greenhouse gas emissions [281,282].

### 7.4. Organic Source Fertilization

Organic farming can enhance carbon sequestration and reduce GHG emissions [283]. The application of 75% organic fertilizer, specifically composted cattle manure, resulted in a substantial increase in the production of summer maize and winter wheat by 15.3–16.7% and 7.2–25.1%, respectively, compared to traditional inorganic fertilization methods. This field experiment was conducted over two consecutive wheat–maize rotation cycles in northern China. The treatments involving 75% and 100% organic fertilizer yielded the lowest nitrous oxide (N_2_O) emissions, achieving reductions of 187.3% and 200.2%, respectively, relative to standard fertilization practices. However, all the fertilizer treatments led to a decrease in methane (CH_4_) absorption by 33.1–82.0% compared to the control. Despite an observed increase in the carbon dioxide flux of 7.7–30.5% during the maize growing season, the combined use of 75% organic and 25% inorganic fertilizer proved to be the most effective strategy. This approach not only maintained high crop yields but also minimized greenhouse gas emissions. Consequently, this integrated fertilization strategy significantly contributed to reducing the global warming potential (GWP) and greenhouse gas intensity (GHGI) within the wheat–maize cropping system [284].

Biochar is described as a prominent strategy in Figure 3, which indicates that its application significantly reduces soil GHG emissions by preventing biochemical decomposition and sequestering GHGs [285]. In contrast, the use of straw and organic matter was found to enhance emissions through increased biochemical decomposition. Notably, after one year, there was a 141.8% increase in soil organic carbon, accompanied by a reduction of 1089.8 kg CO_2_eq ha^−1^ in CO_2_ emissions within a wheat–maize rotation system. These findings underscore the potential of biochar as an organic amendment to decrease GHG emissions and enhance soil carbon sequestration, thereby promoting sustainable agriculture and ecological security [286]. Moreover, the utilization of organic manure has also been associated with improvements in soil organic carbon (SOC); however, it may substantially contribute to greenhouse gas (GHG) emissions, particularly CH_4_ and N_2_O [287]. On the other hand, using biochar is a good strategy, as the application of biochar at a rate of 10 t/ha to maize-cultivated saline–alkali agricultural fields in arid climatic conditions has been demonstrated to be an effective strategy. This practice can enhance the soil organic carbon (SOC) by 46.0%, while concurrently reducing the CO_2_ emissions by 7.0% and N_2_O emissions by 15.0% [288,289]. This underscores the unique characteristics of biochar, notably the stable fixed carbon generated during high-temperature pyrolysis. This process contributes to the reduction of CO_2_ emissions and enhances SOC, while simultaneously promoting enhanced net primary productivity in sorghum fields [290]. Furthermore, a study on biochar demonstrated that the application of biochar significantly reduced the soil N_2_O emissions by 38%, while concurrently increasing the CH_4_ and CO_2_ emissions by 15% and 16%, respectively, as can be seen in Table 3. This leads to an overall decrease in the global warming potential and emission intensity, with achieving an enhancement of the crop yield by 21% [109]. These effects are strongly influenced by the properties of the biochar, soil conditions, and management strategies, highlighting its potential as a climate-smart amendment when optimally utilized [54].

The excessive use of nitrogen does not (necessarily) improve the crop yield. On the contrary, it may lead to increased GHG emissions and nitrate leaching [291]. As multi-cropping systems are increasingly implemented to enhance agricultural yields, they pose various agricultural and ecological challenges due to the excessive application of fertilizers [292,293]. Another approach for boosting the soil health and elevating the crop yield is straw return, which involves re-incorporation of the residual parts of crops into the soil. This technique reduces the pollution that burning straw causes to the environment and influences organic carbon sequestration in the soil [20,294]. Yet, straw incorporation into the soil in linked two distinct processes: the first one is that it attracts soil nutrients (such as nitrogen and carbon substrates) to reduce GHG emissions, while the other is that it supplies carbon and nitrogen bases to soil microorganisms, which can take part in increasing GHG emissions [295], potentially facilitating key soil processes such as decomposition, nitrification, and denitrification [296]. The addition of organic materials, such as biochar and crop residues, provides carbon and nitrogen substrates that stimulate microbial activity [37]. This increased availability of carbon (C) and nitrogen (N) can lead to the expansion of microbial populations and an acceleration of microbial respiration, thereby resulting in elevated carbon dioxide (CO_2_) emissions [37,297]. Moreover, an excess of nitrogen can enhance the denitrification process, leading to increased emissions of nitrous oxide (N_2_O), a potent greenhouse gas [298]. Consequently, while microbial activity is essential for the decomposition of organic matter and the improvement of soil fertility, it can also contribute to increased CO_2_ and N_2_O emissions, particularly when there is an imbalance or excess of available carbon and nitrogen [299]. Furthermore, the decomposition of straw may lead to competition between microbes and crops for nitrogen, potentially resulting in nutrient scarcity, which could impede crop growth and reduce the yield. To mitigate these challenges, it is often recommended to supplement straw return with inorganic nitrogen fertilizers. This combination aims to balance these complex processes to enhance agricultural output, improve soil fertility, and reduce greenhouse gas emissions [300].

The aforementioned strategies are important in lowering GHG emissions while enhancing the production. These strategies, their influencing mechanism and their factors are summarized in Table 4.

## 8. Conclusions

In this review, emissions from both inorganic and organic sources are analyzed, highlighting significant variations in the emission data reported across the literature. Although inorganic nitrogen fertilizer is the primary contributor to greenhouse gas (GHG) emissions, studies indicate that organic sources also contribute to emissions, underscoring the importance of their careful and broader adaptation. To enhance sustainability and mitigate climate impacts, this review emphasizes that adopting integrated agronomic strategies such as optimized nitrogen fertilization, incorporation of organic amendment, modifying irrigation measures, and crop rotation with legumes can significantly reduce GHG emissions. The findings reveal that these mitigation practices influence the GHG dynamics, soil quality, and crop productivity across diverse agroecosystems. Straw return enhances the soil organic matter and microbial activity, thereby promoting carbon sequestration and reducing CO_2_ emissions, particularly when coupled with reduced nitrogen input. Biochar application demonstrates dual benefits: mitigating N_2_O emissions through improved nitrogen retention in soil and enhancing long-term carbon storage. Manure application, although it is a potential source of GHGs as it contains substantial amounts of nitrogen, can significantly contribute to GHG mitigation if properly managed, as it plays a key role in enhancing microbial activity. Modifying nitrogen application to enhance the nitrogen use efficiency (NUE) and soil structure, when applied at optimal rates and timings, results in a net reduction in GHG emissions. Additionally, the implementation of efficient irrigation strategies can contribute to the reduction of GHG emissions, as empirical evidence indicates that the soil moisture significantly influences the regulation of these emissions. In addition, crop rotation with legumes contributes to the natural fixation of nitrogen and reduces the need for synthetic fertilizers, and it promote long-term soil health and sustainability in agricultural system, ultimately reducing GHG emissions. These practices improve the nitrogen use efficiency, maintain soil health, and support climate resilience.

Future studies should concentrate on the long-term impacts of these strategies and evaluate their effects on both soil characteristics and GHG emissions. including the mechanism that is underlying the variations. Therefore, it is recommended that these mitigation strategies should be adopted to reduce the environmental footprint of agriculture while promoting sustainable crop production.

## Figures and Tables

**Table 1:** Types of organic fertilizers with their associated basic properties.

Category	Abbreviation	Type	Constituent/Active Ingredient	Basic Characteristics	References
Straw	S	Crop Residue	Cellulose, Hemicellulose, Lignin, Ash, Protein, Lipids	Carbon Rich, Slowly Decomposes, Improves Soil Structure	[50,51]
Manure	M	Animal Waste	N, P, K, Organic Carbon, Microbial Biomass, Trace Element	Nutrient Rich, High Moisture Content, Improves Soil Fertility	[52,53]
Biochar	BC	Pyrolyzed Organic Matter	Carbon, Ash Content, Microspores, Mesopores	Raises pH in Acidic Soil, Enhances Microbial Activity	[54,55]

**Table 2:** Various fertilization techniques and their impacts on GHG emissions.

Treatment	CO_2_ Emissions (Mg CO_2_-C ha^−1^)	N_2_O Emissions (kg N_2_O-N ha^−1^)	CH_4_ Emissions	Global Warming Potential (GWP)	Other Key Findings	References
Nitrogen (Conv. urea 130 kg N ha^−1^ y^−1^)	2.2 (maize)	0.24 (maize)	-	194 kg CO_2_-eq ha^−1^ yr^−1^	Baseline with conventional urea fertilization.	[68]
Straw Incorporation + CF + compost (SCF, BCF, CF), (CD_20_, CD_50_, Conv. N)	0.77 (fallow)	0.25 (fallow)	144% higher than Conv.	80% higher GWP than conv.	Significant CH_4_ increase during fallow period, but reduced N_2_O emissions in maize.	[68,70]
Biochar + Straw (SCF, BCF, CF)	2.1 (rice–wheat)	0.085 (rice–wheat)	58% reduction in CH_4_	51% lower GWP compared to SCF	Biochar reduces N_2_O and CH_4_ emissions, with enhanced nutrient availability.	[70]
Cow Manure (20% N substitution), Cow Manure (50% N substitution)	2.2 (rice), 2.4 (rice)	0.14 (maize), 0.18 (maize)	-	36.44% higher GWP than conv., 74.58% higher GWP than conv.	Reduces N_2_O emissions by 6.65%, lowers yield by 8.77%	[71]
Biochar (hardwood biochar, fast pyrolysis at 550 °C)	Varies (increase/decrease CO_2_)	63% reduction	Varies (reduction/increase)	-	Reduces N_2_O by up to 63%, with varied impacts on CO_2_ and CH_4_ based on biochar type	[72,73]

**Table 4:** Strategies for GHG emissions reduction and their influence.

Emission Reduction Strategy	Influencing Mechanism	Factors	References
Optimization of fertilizer applications	Denitrification reduction by decreasing excess nitrogen in soil resultantly reduces N_2_O emissions	Crop growth stages Nitrogen levels	[133,244,245]
Manure management	Reduction of methane (CH_4_) and N_2_O emissions by manure via controlling storage and application.	Manure treatment (composting, anaerobic digestion)	[98,274,275]
Conservation tillage	Reducing soil disturbance, improving the storage of soil organic carbon, and lowering N_2_O emissions.	Intensity of tillage (conventional tillage, reduced tillage)	[32,276]
Legume–cereal crop rotation	Improves nitrogen fixation from legumes, which raises nitrogen usage efficiency (NUE).	Crop rotation (soybean–wheat, soybean–maize)	[267,268]
Irrigation	Decreases N_2_O emissions and increases N availability by raising soil moisture.	Irrigation type (deficit irrigation, conventional irrigation)	[249,277,278]

## Data Availability

Data will be provided on reasonable request.

## Abbreviations

The following abbreviations are used in this manuscript:

GHG	Greenhouse gas
N_2_O	Nitrous oxide
CH_4_	Methane
CO_2_	Carbon dioxide
SI	Straw incorporation
N	Nitrogen
NUE	Nitrogen use efficiency
NF	Nitrification
SOC	Soil organic carbon
SOM	Soil organic matter
DNF	Denitrification
VLT	Volatilization
SCF	Straw from the crop into the field
OC	Organic carbon
BCF	Incorporation of biochar from straw into the field
CF	Incorporation of chemical fertilizer alone into the field
CONV	Conventional urea
CD_20_	Cow manure at 20.00%
CD_50_	Cow manure 50.00%
DN600	Drip fertigation treatments, 600 kg N ha^−1^ yr^−1^
Biochar	Slow pyrolysis of sugarcane straw at 450 °C with heating rate of 10 °C min^−1^ and a retention time of 2 h.
CS	Single application of 13.5 t/ha biochar (CS)
AM	Animal manure (pig manure) application rate of 15 Mg ha^−1^ yr^−1^
CRM	Solid cattle manure at a constant, CRM; 45 Mg ha^−1^
VRM	Variable rate manure application, VRM; 0–72 Mg ha^−1^
N-enriched	114 mL of liquid ammonium-based N-fertilizer (N-enriched)
CsM	Solid cattle manure
PM	Poultry manure
